# Effect of Organic Diet Intervention on Pesticide Exposures in Young Children Living in Low-Income Urban and Agricultural Communities

**DOI:** 10.1289/ehp.1408660

**Published:** 2015-04-10

**Authors:** Asa Bradman, Lesliam Quirós-Alcalá, Rosemary Castorina, Raul Aguilar Schall, Jose Camacho, Nina T. Holland, Dana Boyd Barr, Brenda Eskenazi

**Affiliations:** 1 Center for Environmental Research and Children’s Health (CERCH), School of Public Health, University of California, Berkeley, Berkeley, California, USA; 2 Maryland Institute for Applied Environmental Health, School of Public Health, University of Maryland, College Park, Maryland, USA; 3 Rollins School of Public Health, Emory University, Atlanta, Georgia, USA

## Abstract

**Background:**

Recent organic diet intervention studies suggest that diet is a significant source of pesticide exposure in young children. These studies have focused on children living in suburban communities.

**Objectives:**

We aimed to determine whether consuming an organic diet reduced urinary pesticide metabolite concentrations in 40 Mexican-American children, 3–6 years of age, living in California urban and agricultural communities.

**Methods:**

In 2006, we collected urine samples over 16 consecutive days from children who consumed conventionally grown food for 4 days, organic food for 7 days, and then conventionally grown food for 5 days. We measured 23 metabolites, reflecting potential exposure to organophosphorous (OP), pyrethroid, and other pesticides used in homes and agriculture. We used linear mixed-effects models to evaluate the effects of diet on urinary metabolite concentrations.

**Results:**

For six metabolites with detection frequencies > 50%, adjusted geometric mean concentrations during the organic phase were generally lower for all children, and were significant for total dialkylphosphates (DAPs) and dimethyl DAPs (DMs; metabolites of OP insecticides) and 2,4-D (2,4-dichlorophenoxyacetic acid, a herbicide), with reductions of 40%, 49%, and 25%, respectively (*p* < 0.01). Chemical-specific metabolite concentrations for several OP pesticides, pyrethroids, and herbicides were either infrequently detected and/or not significantly affected by diet. Concentrations for most of the frequently detected metabolites were generally higher in Salinas compared with Oakland children, with DMs and metolachlor at or near significance (*p* = 0.06 and 0.03, respectively).

**Conclusion:**

An organic diet was significantly associated with reduced urinary concentrations of nonspecific dimethyl OP insecticide metabolites and the herbicide 2,4-D in children. Additional research is needed to clarify the relative importance of dietary and non-dietary sources of pesticide exposures to young children.

**Citation:**

Bradman A, Quirós-Alcalá L, Castorina R, Aguilar Schall R, Camacho J, Holland NT, Barr DB, Eskenazi B. 2015. Effect of organic diet intervention on pesticide exposures in young children living in low-income urban and agricultural communities. Environ Health Perspect 123:1086–1093; http://dx.doi.org/10.1289/ehp.1408660

## Introduction

Although most residential uses of many organophosphorus (OP) pesticides, including chlorpyrifos and diazinon, have been phased out since the mid-2000s due to potential health risks to children, they have continued to be used in agriculture [U.S. Environmental Protection Agency (EPA) 2000, 2001]. The use of OP pesticides in agriculture could result in ingestion of residues in food, and recent studies suggest that dietary intake of produce and juices may account for a significant proportion of OP pesticide exposure in young children (Lu et al. 2006b, 2008; Morgan et al. 2005; Smith-Spangler et al. 2012; Wilson et al. 2003). Some of the best evidence supporting these findings includes results from diet intervention studies where significant reductions in excreted urinary pesticide metabolites were observed in young children when they consumed an organic diet (Lu et al. 2006b, 2008). For example, Lu et al. (2008) showed that several OP pesticide metabolites in suburban children declined to undetectable concentrations during several days of eating organic food. These findings were consistent with an earlier observational study, which suggested that children consuming primarily organic food in non-agricultural households with no residential pesticide use have minimal or no pesticide exposures (Curl et al. 2003). Lu et al. (2006a, 2009) also reported a decrease in urinary pyrethroid pesticide metabolite concentrations in these children during the organic diet phase. The lower urinary pesticide metabolite concentrations found in children eating organic diets is consistent with food residue monitoring data that has shown lower pesticide residue levels in organic versus conventionally grown food [Baker et al. 2002; U.S. Department of Agriculture (USDA) 2008].

Other factors associated with children’s cumulative pesticide exposures include socioeconomic status and location of residence. For example, low-income children may experience higher exposures to pesticides, particularly pyrethroids, because of poor housing quality and associated pest infestations and home pesticide use (Bradman et al. 2005a; Quirós-Alcalá et al. 2011; Whyatt et al. 2002). Children living in agricultural areas, compared with children living in non-agricultural suburban areas, are exposed to higher ambient and residential contamination from drift or volatilization from nearby agricultural applications and take-home residue by farmworking parents (Bradman et al. 2011; Harnly et al. 2009; Lu et al. 2000; Quirós-Alcalá et al. 2011).

To date, organic food intervention studies have been conducted only in suburban/non-agricultural communities. Therefore, the relative contribution of dietary versus non-dietary pesticide exposures in low-income children living in urban and agricultural communities is not known. To address existing data gaps, we conducted an organic diet intervention study in young, low-income Mexican-American children living in urban and agricultural communities. We report results for 23 urinary metabolites of OP and pyrethroid insecticides, as well as several herbicidal compounds.

## Methods

*Study participants*. We recruited a convenience sample of 40 children, 20 residing in an urban community in the Fruitvale area of Oakland, California, and 20 residing in a predominantly agricultural community in Salinas, California. Eligible families had a child who was between 3 and 6 years of age, was toilet trained, and normally consumed conventional (non-organic) foods. For participating Salinas families, at least one household resident worked in agriculture. To minimize cultural disparities in diet between children living in both locations, eligible children were Mexican immigrants or Mexican American. Participants were recruited through local community clinics and organizations primarily serving low-income populations. All sampling was completed between July and September 2006. The University of California, Berkeley, Committee for the Protection of Human Subjects reviewed and approved all study protocols, and written informed consent was obtained from parents before data collection.

**Figure 1:**
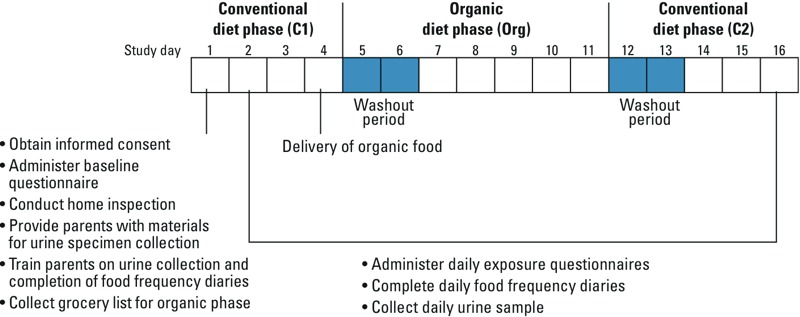
Study activities by day.

*Data collection*. Families participated in the study for 16 consecutive days. On the first day, bilingual staff obtained consent; administered a baseline questionnaire to collect information on household characteristics and pesticide exposure behaviors (e.g., recent pesticide use at home or workplace); conducted a home inspection to record information on pest infestations, pesticide active ingredients, and proximity to agricultural fields; provided materials for urine specimen collection; and trained parents on how to collect urine specimens and complete child food intake diaries. Parents also submitted a grocery list for food items to be consumed during the organic diet phase, and the food was delivered to the family on the fourth day. Parents recorded all of the food items and portion size consumed by the child each day based on validated guidelines (Block et al. 1990, 1992). Staff conducted daily in-person interviews with the mother when they picked up the urine specimen and the food intake diaries. The interview collected information on home and workplace pesticide use and on the child’s compliance with the diet protocols (i.e., parents were asked if the child ate food outside of the home or any conventional foods during the organic diet phase and whether they consumed any leftover organic food during the second conventional diet phase) (Figure 1 shows timing of study activities). Parents were provided with gift certificates to local grocery stores for their participation in the study and children were provided with educational materials/toys at five different time points throughout the study to encourage adherence to the diet protocol and provision of urine samples.

*Diet protocol*. Children followed a conventional diet for the first 4 days (conventional phase 1; C1), then an organic diet for 7 days, and then returned to a conventional diet phase for the remaining 5 days (conventional phase 2; C2). For the organic diet phase, parents were instructed to request food items that were normally consumed by the participating child to ensure that any observed changes in urinary metabolite concentrations would not be attributed to changes in diet. Organic foods provided included fruits, breads, cereals, vegetables, dairy, eggs, juices, and snack foods. To facilitate adherence to the organic diet phase, enough food was provided for the entire family. Most of the organic food provided was purchased from the same grocery store chain in both communities. Parents recorded child dietary consumption habits in food frequency diaries throughout the conventional and organic diet phases (see Supplemental Material, Table S1).

*Urine specimen collection*. Parents collected children’s first morning voids for 15 consecutive days starting on the second day of the study (i.e., days 2–16; Figure 1). If the parents missed the first morning void, they were instructed to collect the next spot urine sample. Children were given the option of voiding directly into a collection jar or a Specipan™ (Baxter Scientific, McGaw Park, IL). If voids were collected in Specipans™, parents transferred the specimen into collection jars. Parents recorded the collection time of each void and stored specimens in a portable refrigerator provided by study staff. Study staff collected the specimen and provided parents with new collection materials for the next collection. Samples were aliquoted and stored at –80°C until shipped on dry ice to the Centers for Disease Control and Prevention (CDC), National Center for Environmental Health in Atlanta, Georgia, for laboratory analysis. For quality control purposes, frozen field blanks and spikes prepared by CDC were thawed, re-packaged to blind the samples to the analyst, and shipped to the laboratory with the study specimens. We collected a total of 594 urine samples, most of which were first morning voids (> 90%). Except for one child who dropped out of the study on the 8th day, each child provided at least 14 urine samples for laboratory analysis.

*Laboratory analysis of urine specimens*. We measured 23 pesticide metabolites in urine specimens including specific and nonspecific metabolites for OP, pyrethrin, and pyrethroid insecticides and select herbicides (Table 1 lists precursor compounds). Selection of metabolites was based on use of precursor compounds in the study locations at the county level, potential for pesticide exposure from residential and agricultural applications, and the availability of validated laboratory methods. We measured six dialkylphosphate (DAPs) metabolites, including diethyls (DEs) [diethylphosphate (DEP), diethylthiophosphate (DETP), and diethyldithiophosphate (DEDTP)] and dimethyls (DMs) [dimethylphosphate (DMP), dimethylthiophosphate (DMTP), and dimethyldithiophosphate (DMDTP)]. These nonspecific urinary OP pesticide metabolites derive from approximately 28 OP insecticides registered for use by the U.S. EPA, many of which are applied for agricultural and non-agricultural purposes (Table 1) in the participating communities. We also measured five specific OP pesticide metabolites: CMH (coumaphos), IMPY (diazinon), CIT (isazophos), MDA (malathion), and DPY (pirimiphos-methyl). Other insecticide metabolites measured included two specific pyrethroid metabolites [4FP (cyfluthrin) and DBCA (deltamethrin)] and four nonspecific pyrethroid metabolites (3-PBA, CDCA, and *cis*-/*trans*-DCCA). Specific metabolites of six herbicides were also measured [2,4-D (2,4-dichlorophenoxyacetic acid), 2,4,5-T (2,4,5-trichlorophenoxyacetic acid), acetochlor, alachlor, atrazine, and metolachlor], three of which (alachlor, atrazine, and metolachlor) were used only in the agricultural study location. All 594 samples were analyzed for DAP metabolites, whereas the number of samples analyzed for specific metabolites ranged between 532 and 594, depending on the amount of urine volume remaining after initial DAPs analysis.

**Table 1:** Summary of precursor compounds, including reported usage by county, and measured metabolites in urine.

Chemical class and precursor compounds	Metabolite measured (abbreviation)	Precursor compound use in 2006 (kg)^*a,b,c*^		LOD (ng/mL)	Overall analyte DF (%)^*d*^
		Monterey	Alameda		
Organophosphorus insecticides
Coumaphos	3-Chloro-4-methyl-7-hydroxycoumarin (CMH)	0	0	0.18	23
Diazinon	2-Isopropyl-4-methyl-6-hydroxyprimidin (IMPY)	65,813	5	0.1	23
Isazophos	5-Chloro-1,2-dihydro-1-isopropyl-[3H]-1 (CIT)	0	0	0	31
Malathion	Malathion dicarboxylic acid (MDA)	16,940	8	0.05	38
Pirimiphos-methyl	2-Diethylamino-6-methylpyrimidin-4-ol (DPY)	0	0	0.2	4
Azinphos-methyl, chlorpyrifos-methyl, dichlorvos, dicrotophos, dimethoate, fenitrothion, fenthion, isazofos-methyl, malathion, methidathion, methyl parathion, naled, oxydemeton-methyl, phosmet, pirimiphos-methyl, temephos, tetrachlorvinphos, trichlorfon	Total dimethylphosphates (total DMs = DMP + DMTP + DMDTP)	75,447	624	DMP: 0.6 DMTP: 0.2 DMDTP: 0.1	92
Chlorethoxyphos, chlorpyrifos, coumaphos, diazinon, disulfoton, ethion, parathion, phorate, sulfotepp, terbufos	Total diethylphosphates (total DEs = DEP + DETP + DEDTP)	95,812	73	DEP: 0.2 DETP: 0.1 DEDTP: 0.1	74
Totals	Total dialkylphosphates (total DAPs = total DMs + total DEs)	171,259	697	—	95
Pyrethrin and pyrethrin insecticides
Cyfluthrin	4-Fluoro-3-phenoxybenzoic acid (4FP)	84	392	0.12	29
Deltamethrin	*cis*-2,2-(Dibromo)-2-dimethylvinylcyclopropane carboxylic acid (DBCA)	17	67	0.3	0.8
Allethrin, phenothrin, prallethrin, pyrethrins, resmethrin, tetramethrin	Chrysanthemum dicarboxylic acid (CDCA)	112	27	0.21	ND
Allethrin, cyhalothrin, cypermethrin deltamethrin, fenpropathrin, permethrin, tralomethrin	3-Phenoxybenzoic acid (3-PBA)	15,631	1,008	0.2	82
*cis*-Cypermethrin, *cis*-cyfluthrin, *cis*-permethrin	*cis*-2,2-(Dichloro)-2-dimethylvinylcyclopropane carboxylic acid (*cis*-DCCA)	13,180	1,287	0.3	0.8
*trans*-Cypermethrin, *trans*-cyfluthrin, *trans*-permethrin	*trans*-2,2‑(Dichloro)‑2‑dimethylvinylcyclopropane carboxylic acid (*trans*-DCCA)	13,180	1,287	0.3	7
Herbicides
2,4-Dichlorophenoxyacetic acid	2,4-Dichlorophenoxyacetic acid (2,4-D)	0	0	0.1	90
2,4,5-Trichlorophenoxyacetic acid	2,4,5-Trichlorophenoxyacetic acid (2,4,5-T)	0	0	0.1	23
Acetochlor	Acetochlor mercapturate (ACE)	0	0	0.21	7
Alachlor	Alachlor mercapturate (ALA)	46	0	0.3	25
Atrazine	Atrazine mercapturate (ATZ)	65	0	0.27	ND
Metolachlor	Metolachlor mercapturate (MET)	1,550	0	0.1	72
Abbreviations: DF, detection frequency; LOD, limit of detection; ND, not detected. ^***a***^California EPA Department of Pesticide Regulation (Cal/EPA DPR) Pesticide Use Reporting (PUR) Database (CalEPA DPR 2006a, 2006b). ^***b***^Amount applied (kg) includes agricultural, landscape maintenance, and structural pest control uses reported in each county; by law all agricultural pesticide use (includes applications to parks, golf courses, cemeteries, rangeland, pastures, and along roadside and railroad rights-of-way) must be reported to the CalEPA DPR. The PUR database does not provide amount applied for each individual permethrin isomer (*cis*-, *trans*-); formulations applied may consist of a combination of two or more isomers. ^***c***^The following insecticides were not applied in Monterey or Alameda County in 2006: Chlorpyrifos-methyl, dichlorvos, dicrotophos, fenitrothion, fenthion, isazophos-methyl, pirimiphos-methyl, temephos, tetrachlorvinphos, trichlorfon, chlorethoxyphos, coumaphos, ethion, parathion, sulfotepp, and terbufos. ^***d***^Overall analyte DF (%) is the ratio of the total number of urine samples with analyte concentration > LOD during the entire study to the total number of urine samples collected in the study multiplied by 100.					

Laboratory methods used to measure urinary DAPs have been described previously (Bravo et al. 2004). Briefly, we lyophilized specimens to remove water and redissolved the residue in acetonitrile:diethyl ether. We then derivatized DAPs to their chloropropyl phosphate esters, and concentrated extracts were analyzed by isotope dilution gas chromatography tandem mass spectrometry. Quantification of specific OP, pyrethroid, and herbicide metabolite concentrations was performed using a method described previously (Olsson et al. 2004). Solid-phase extraction was used to extract samples, and analysis of samples was performed on high-performance liquid chromatography (Agilent 1100; Agilent Technologies, Waldbronn, Germany) coupled with tandem mass spectrometry, using a triple quadrupole mass spectrometer (TSQ 7000; ThermoFinnigan, San Jose, CA) with an atmospheric pressure ionization interface for analyzing the OP pesticide metabolites and herbicides. A triple quadrupole mass spectrometer (Sciex API4000; Applied Biosystems/MDS Sciex, Foster City, CA) was used for detecting the pyrethrin and pyrethroid metabolites.

Quality control (QC) procedures included repeat analysis of three in-house urine pools enriched with known amounts of metabolite residues with target values and confidence limits that were previously determined. Westgard rules for quality control were used to validate each analytical run (Caudill et al. 2008). We also analyzed duplicate samples within runs of the same sample (typically one sample per run, or about 5% replicates) to assess the precision of our analytical runs. These values were always within ± 20% or else the samples were repeated. No metabolites were present in any blank samples, indicating that no contamination occurred in the field, during sample processing, or during shipment to the laboratory. Recoveries of repeat QC samples were 100 + 10% and relative standard deviations were below 15%. Limits of detection (LOD) ranged between 0.05 and 0.30 ng/mL (Table 1). Metabolite concentrations below the LOD were imputed to LOD divided by the square root of 2 (Hornung and Reed 1990). Because individual OP pesticides can devolve to more than one DAP metabolite, we summed the DAPs on a molar basis to reflect total DM (i.e., molar sum of DMP, DMTP, and DMDTP) and total DE (i.e., molar sum of DEP, DETP, and DEDTP) metabolites. Total DAPs consisted of the molar sum of total DMs and total DEs. Creatinine concentrations were determined using a commercially available method (Vitros CREA slides; Ortho Clinical Diagnostics, Raritan, NJ).

*Data analysis*. We first summarized demographic characteristics for participating children. Before data analysis, we excluded urine samples collected during days 5 and 6 (considered washout days between the first conventional diet phase and the organic diet phase) and during days 12 and 13 (considered washout days between the organic diet phase and the second conventional diet phase) from our analysis (Figure 1). We also excluded urine samples from children who reported eating at a restaurant or eating at a friend’s house on the preceding day during the organic diet phase, or eating organic food on the preceding day during the second conventional diet phase. Additionally, if the family reported pesticide use in or around the home during the study period, then observations for metabolites of the active ingredients in the formulation applied were excluded for the day of and day after pesticide application. If the active ingredients were unknown, then observations were excluded for all metabolites. Last, we excluded pyrethroid metabolite results for one child in Salinas with mean 3-PBA concentrations > 3 SDs away from the mean of the other children. Based on the aforementioned exclusions, the total number of urine samples included in our final statistical models varied for each metabolite and ranged from 331 to 398 samples. Final statistical models focused on metabolites with an overall detection frequency in urine of ≥ 50%. Metabolite concentrations were log_10_-transformed for statistical analyses.

We computed descriptive statistics for frequently detected metabolites [*n* = 6; total DMs, total DEs, total DAPs, metolachlor mercapturate (MET), 2,4-D, 3-PBA] by diet phase. We then used linear mixed-effects models to account for the correlation among repeat urine samples collected from the same individual and determined whether mean metabolite concentrations differed between the organic diet phase and each of the conventional diet phases separately. These comparisons were conducted for all children and then stratified by location. To control for the large number of statistical tests (i.e., 36 multiple comparisons), we used the Hochberg procedure (Hochberg 1988).

We then conducted analyses using adjusted linear mixed-effects models to evaluate the effect of an organic diet on children’s urinary metabolite concentrations controlling for location of residence and whether the sample was a first morning void or not. We allowed only for random intercepts, and the covariance matrix was assumed to have identical variances and all covariances equal to zero. Effects from location, diet, and first morning void were fixed. The variance–covariance matrix of the parameter estimates was calculated using the robust sandwich estimator. For these analyses, we defined a binary diet variable, which combined all urine samples from both conventional diet phases into one category and the samples from the organic diet phase into another because no statistically significant differences were observed between metabolite concentrations in C1, the first conventional diet phase, compared with C2 (not shown). We also assessed whether the effect of the organic diet differed by location by including an interaction term; presence of interaction was established at *p* < 0.20. If the interaction term was not significant, we ran the model without it for our estimations. In those cases where interaction between location and diet was present, we used postestimation procedures to obtain the marginal effects of the organic diet for children at each location and the overall average marginal effect for all children. Unless otherwise noted, significance was set at an adjusted *p* < 0.05.

Additionally, we used the Kruskal–Wallis test to examine whether the number of servings of different food categories (e.g., vegetables, fruits) changed during the three diet phases. If this test suggested a significant difference, we then performed a pairwise Wilcoxon rank sum test to determine which diet phases differed.

For statistical analyses, we present results that are not adjusted for creatinine, to be consistent with other studies (Barr et al. 2005; Bradman et al. 2011; Lu et al. 2008). Analyses were repeated with creatinine-adjusted values to confirm our bivariate results. In addition, we performed all multivariate models with creatinine-adjusted urinary metabolite concentrations for comparison with final models.

## Results

*Demographic characteristics*. Forty children 3–6 years of age participated in this study: 19 boys and 21 girls. Demographic characteristics were similar between study locations (see Supplemental Material, Table S2). The mean (± SD) age for children was 4.5 ± 1.1 years and 4.8 ± 1.2 years in the urban (Oakland, *n* = 20; 10 boys and 10 girls) and agricultural community (Salinas, *n* = 20; 9 boys and 11 girls), respectively. The majority of participants in both communities (65%) were within 200% of the U.S. federal poverty threshold, and almost all parents (95%) reported that they were married or living as married. Most homes (> 80%) in each community were located > 0.25 mile from the nearest agricultural field or golf course (homes in the agricultural community were located in East Salinas, generally removed from agricultural fields); four homes in Salinas were located < 0.25 mile from the nearest agricultural field. Overall, 23% of participants reported home pesticide use during the study period (30% and 15% of urban and farmworker households, respectively). However, participants in the urban community of Oakland reported more home pesticide use in the 3 months preceding the study compared with participants in the farmworker community of Salinas (65% vs. 30%, respectively).

*Frequency of food consumption*. Information collected in food frequency diaries indicated that overall dietary consumption habits did not change during the different diet phases (see Supplemental Material, Table S1). Fruit and grain consumption, however, was higher in the organic diet phase compared with the conventional diet phases (*p* < 0.05).

*Pesticide metabolite levels in urine*. Of the 13 pesticide-specific metabolites measured, 2 had overall detection frequencies (DFs) > 50%: 2,4-D at 90% and MET at 72% (Table 1). The nonspecific pyrethroid metabolite 3-PBA had an overall DF of 82%. Overall DFs for total DMs, DEs, and DAPs were 92%, 74%, and 95%, respectively. Thus, all but 6 metabolites measured in this study had DFs < 50%. The distributions of these 6 metabolites are presented in the Supplemental Material, Table S3. The DFs during conventional and organic diet phases for metabolites not frequently detected are presented in the Supplemental Material, Table S4.

*Results of bivariate analyses*. Among all children (*n* = 40), the geometric mean (GM) of total DMs, DAPs, and 2,4-D decreased during the organic diet phase by –48.6% [95% confidence interval (CI): –63.2, –25.8%; *p* < 0.001], –39.7% (95% CI: –55.8, –13.3%; *p* = 0.005), and –21.0% (95% CI: –35.3, –1.9%; *p* = 0.03), respectively, compared with C1, the first conventional diet phase. For example, the GMs (GSDs) for total DMs, DAPs, and 2,4-D were 105.4 (3.8), 149.6 (3.4), and 0.4 (2.6), respectively, during C1 compared with 54.1 (4.3), 90.2 (4.0), and 0.3 (2.2), respectively, during the organic diet phase. Similarly, the GMs of total DMs, DAPs, and 2,4-D were considerably lower during the organic diet phase compared with C2, the second conventional diet phase [–52.1% (95% CI: –68.5, –24.9%; *p* = 0.001), –45.3% (95% CI: –62.6, –15.7%; *p* = 0.005), and –32.8% (95% CI: –45.4, –14.1%; *p* = 0.001), respectively]. After adjusting for multiple comparisons, only those results with *p* < 0.001 remained significant. Because total DM concentrations were consistently much higher than DE concentrations, the findings for total DAPs were driven by total DMs, which break down from insecticides such as oxydemeton-methyl and malathion. Concentrations of total DEs, MET, and 3-PBA were not significantly affected by diet. No statistically significant differences were found between metabolite levels in C1 compared with C2 (see Supplemental Material, Table S3).

**Figure 2:**
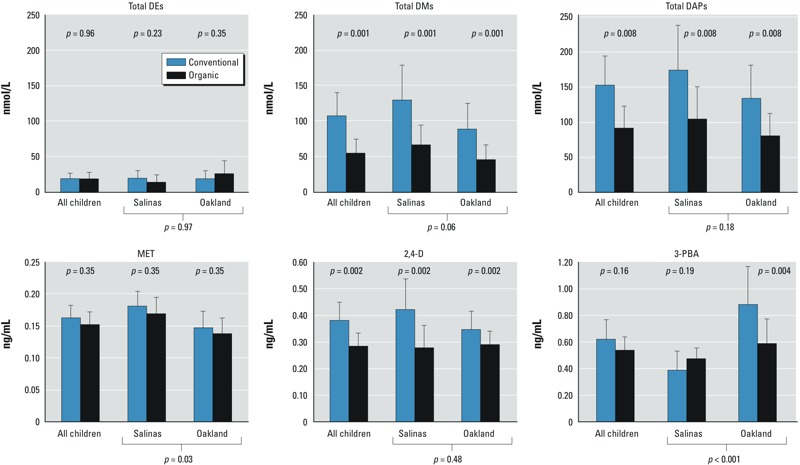
Estimated marginal adjusted GMs and 95% CIs for select urinary metabolites based on diet followed after fitting of linear mixed-effects models. All models were adjusted for type of void (first morning void vs. random spot sample). Models for “All children” were also adjusted for location (Oakland vs. Salinas); an interaction term for location and diet was included in these models for total DEs and 3-PBA (*p*_interaction _≤ 0.20). *p*-Values reported in the figure indicate whether there were significant differences observed in metabolite concentrations between diet phases by location. *p*-Values reported in the *x*-axis indicate significance for the difference in metabolite concentrations between locations regardless of diet.

*Estimated effect of diet on urinary metabolite levels using multivariate models*. All children. Results from multivariate models showed significantly lower metabolite concentrations of total DMs (–48.7%; 95% CI: –65.7, –23.2%), total DAPs (–39.9%; 95% CI: –58.6, –12.6%), and 2,4-D (–25.2%, 95% CI: –38.0, –9.7%) (*p* < 0.01) during the organic diet phase compared with the combined conventional diet phases (Table 2 and Figure 2). We also observed lower metabolite concentrations during the organic diet phase, albeit not significantly, for total DEs, MET, and 3-PBA.

**Table 2:** Estimated effect of an organic diet (vs. conventional) on the geometric mean for frequently detected metabolites.*^a^*

Children	Total DEs	Total DMs	Total DAPs	MET	2,4-D	3-PBA
All (*n *= 40)
Percent change (95% CI)	–1.2 (–35.3, 51.0)	–48.7 (–65.7, –23.2)	–39.9 (–58.6, –12.6)	–6.4 (–18.6, 7.5)	–25.2 (–38.0, –9.7)	–13.3 (–28.9, 5.8)
*p*-Value	0.957	0.001	0.008	0.350	0.002	0.159
Oakland (*n *= 20)
Percent change (95% CI)	36.4 (–29.0, 162.0)	—	—	—	—	–32.7 (–48.8, –11.7)
*p*-Value	0.352					0.004
Salinas (*n *= 20)
Percent change (95% CI)	–28.1 (–58.0, 23.1)	—	—	—	—	21.5 (–8.9, 62.0)
*p*-Value	0.229					0.185
*p*_Interaction_	0.137	0.393	0.229	0.498	0.209	0.003
Abbreviations: CI, confidence interval. ^***a***^Marginal results by location are omitted if observed interaction between location and diet was not significant (*p* > 0.2). In these cases only the model without interaction term is presented for all children.						

Except for 2,4-D, the linear mixed-effects model results using creatinine-adjusted metabolites were similar to models with metabolites not adjusted for creatinine (see Supplemental Material, Table S5 and Figure S1).

Among the six frequently detected metabolites, we observed significant interactions (*p* < 0.20) between location and diet only for total DEs and 3-PBA (see Table 2). Analyses evaluating the effect of location are described below.

Oakland children. For children living in Oakland, switching from a conventional diet to an organic diet had the same effect on mean total DM, total DAP, and 2,4-D levels as observed for all children, because there was no interaction between diet and location for these compounds (results presented above). Switching to an organic diet was associated with a –32.7% (95% CI: –48.8, –11.7%) decrease in mean 3-PBA levels (*p* = 0.004) among Oakland children. The opposite effect was observed for mean concentrations of total DEs, where an increase of 36.4% (95% CI: –29.0, 162.0) was observed during the organic diet phase. However, this large relative change reflected small absolute differences and was nonsignificant (*p* = 0.352) (Table 2 and Figure 2).

Salinas children. For children living in Salinas, the effect of switching from a conventional to an organic diet on the mean concentrations of total DMs, total DAPs, and 2,4-D metabolites was again the same as for all children because of the lack of interaction with location (results presented above). Contrary to what was observed in Oakland, among Salinas children, switching from a conventional to an organic diet was associated with a 21.5% (95% CI: –8.9, 62.0) increase in mean 3-PBA levels and a –28.1% (95% CI: –58.0, 23.1) decrease in total DEs. Neither of these changes, however, was statistically significant (*p* = 0.185 and 0.229, respectively) (Table 2 and Figure 2).

Salinas versus Oakland children. Among the six metabolites with detection frequencies > 50%, we found that concentrations were higher, with the exception of 3-PBA, in Salinas children compared with Oakland children, regardless of diet (*p*-values displayed on Figure 2). We observed somewhat higher adjusted GM concentrations for DM metabolites in Salinas versus Oakland children: GM = 97.7 (95% CI: 75.04, 127.09) versus GM = 66.9 (95% CI: 49.90, 89.67), respectively (*p* = 0.06); and significantly higher MET concentrations in Salinas versus Oakland children: GM = 0.18 (95% CI: 0.16, 0.20) versus GM = 0.14 (95% CI: 0.13, 0.17), respectively (*p* = 0.03). Conversely, 3-PBA concentrations were significantly higher in Oakland than in Salinas children: GM = 0.76 (95% CI: 0.60, 0.97) versus GM = 0.42 (95% CI: 0.34, 0.52), respectively (*p* < 0.001).

## Discussion

Metabolites representing several classes of pesticides, including OP and pyrethroid insecticides and the herbicides 2,4-D and metolachlor, were frequently detected (> 72%) in urine samples collected from participating children. Multivariate analyses indicated that consuming an organic diet significantly lowered urinary concentrations of total DMs, total DAPs, and 2,4-D for children in both rural and urban locations. Among other frequently detected analytes, nonspecific diethyl OP pesticide metabolites and the pyrethroid metabolite 3-PBA, as well as MET, were not significantly lower for all children during the organic diet phase. Specific metabolites for other pesticides used on produce or grain were not frequently detected, including metabolites of the OP insecticides diazinon, malathion, and pirimiphos and the herbicides 2,4,5-T, acetochlor, and atrazine.

Similar findings of significantly reduced total DM and DAP metabolite levels during an organic diet phase were recently reported in a crossover study of 13 adults living in Melbourne, Australia (Oates et al. 2014). Results from that study showed that mean total DAP metabolite levels were 89% lower during the 1-week organic diet phase compared with the conventional diet phase. The researchers (Oates et al. 2014) found an even greater reduction (96%) for total DMs. In addition, Curl et al. (2015) recently found that urinary DAP concentrations were significantly lower among participants in the Multi-Ethnic Study of Atherosclerosis (MESA) who reported more frequent consumption of organic produce (*p* < 0.02). Our finding that DAP metabolite levels (i.e., nonspecific OP pesticide metabolites) declined during the organic diet phase is also consistent with findings for specific metabolites by Lu et al. (2006b, 2008) showing that fruit and vegetable consumption is associated with OP pesticide–specific urinary metabolite levels in children. As total urinary DM concentrations were consistently much higher than DE concentrations, findings for total DAPs in our study were driven by total DMs, which break down from insecticides such as azinphos-methyl, commonly used on tree-fruit crops consumed by children. In previous studies, we have observed much higher variability and instability of DE metabolites (Bradman et al. 2007, 2013), which may have limited our power to examine the effect of diet on total DE metabolite concentrations.

Our finding that an organic diet was not associated with a significant reduction in pyrethroid metabolite (3-PBA) excretion for all children is not surprising given that these pesticides are primarily used in and around homes and not commonly applied to food crops; the finding is also consistent with Lu et al. (2006a), who reported that residential use is a more significant pyrethroid exposure factor for children than a conventional diet. Although we did observe a significant decrease in 3-PBA concentrations for Oakland children, we did not observe a significant reduction in metabolite levels for Salinas children during the organic diet phase. In contrast to prior organic diet intervention studies (Lu et al. 2006b, 2008), MDA detection frequencies were low in our population, and it was not possible to examine trends related to diet.

Several studies indicate that dietary intake is a potential route of exposure for herbicides. For example, Morgan et al. (2008) detected 2,4-D in approximately 46% of composite food samples at concentrations up to 20 ng/g. Similarly, Wilson et al. (2003) detected 2,4-D in 100% of liquid food samples and 89% of solid food samples, and estimated that dietary ingestion accounted for approximately 94% of total 2,4-D exposure in young children from combined dietary and nondietary ingestion and inhalation. In the 2003–2005 Food and Drug Administration’s Total Diet Study (FDA 2003, 2005), the FDA detected 2,4-D in only a few types of cereals and grains; however, their laboratory detection limits were higher than other studies. Overall, these studies indicate that 2,4-D may be present in food and support our finding that the lower levels observed in our population during the organic diet phase were attributable to lower dietary exposure.

Herbicides such as metolachlor and atrazine have been tested in a wide variety of foods with very low detection frequencies (USDA 2007, 2013). Limited testing for other herbicides including alachlor and acetochlor also shows low detection frequencies in food (USDA 2007, 2013). Although many of these herbicides have been frequently detected in drinking water, the monitoring data suggest that solid foods are a less important source of exposure to these compounds (USDA 2007, 2013), consistent with our finding that organic diet was not associated with significantly reduced excretion of metabolites for these compounds. All of the herbicides for which we measured metabolites (2,4-D, 2,4,5-T, acetochlor, alachlor, atrazine, and metolachlor) had relatively low or no agricultural use in the regions we studied (Table 1) [California Environmental Protection Agency, Department of Pesticide Regulation (CalEPA DPR) 2006a, 2006b], and incidence of water contamination in our study regions is rare (CalEPA DPR 2006c).

The higher pyrethroid metabolite 3-PBA concentrations we observed in the Oakland children compared with Salinas were consistent with reported higher recent use of home pesticides, as well as higher pyrethroid pesticide residues that we measured previously in these children’s homes (Quirós-Alcalá et al. 2011). Concentrations of metolachlor were higher in Salinas children compared with Oakland children regardless of diet. This finding is consistent with zero use of metolachlor reported in Alameda County (where the Oakland homes are located) (Table 1) compared with 1,550 kg applied in Monterey County (where Salinas homes are located). The somewhat higher levels of DMs in Salinas than in Oakland children is consistent with our results showing primarily higher levels of DMs than of DEs in Salinas pregnant women participating in the CHAMACOS (Center for the Health Assessment of Mothers and Children of Salinas) study compared with women of reproductive age in NHANES (Bradman et al. 2005b).

Overall, we found few differences in food choices between the conventional and organic diet phases in this study. However, there appeared to be slightly higher intake of fruit and grains among participants during the organic diet phase (see Supplemental Material, Table S1). These differences are unlikely to have confounded our results. For example, several studies have shown that higher produce intake is associated with higher urinary pesticide metabolite levels (Curl et al. 2015; Lu et al. 2005, 2006b; Oates et al. 2014). Thus, without the organic diet, we would expect that more fruit in general would lead to higher urinary metabolite levels, the opposite of what we found for several metabolites.

This study has several limitations. Urinary metabolite concentrations for some insecticides (e.g., DAPs) may reflect exposure to precursor pesticide compounds or preformed metabolites in food or the environment (Lu et al. 2005; Quirós-Alcalá et al. 2012; Zhang et al. 2008). Thus, reductions in urinary metabolite levels during the organic diet phase may, in part, be attributable to reduced intake of preformed metabolites from eating organic food, which presumably has fewer preformed metabolites because it was not treated with pesticides that can further breakdown. However, food monitoring data indicate that conventional foods have significantly higher pesticide residues compared with organic food (Baker et al. 2002; Forman et al. 2012; USDA 2008), suggesting that the reductions in child pesticide urinary metabolites during organic diet phases are at least partly attributable to reductions in precursor pesticide exposure. Given that OP urinary metabolite levels, especially in pregnant women, have been associated with poorer neurodevelopment in children (Bouchard et al. 2011; Muñoz-Quezada et al. 2013), future research clarifying the contribution of preformed metabolites to human exposure is critically needed to inform exposure and risk assessment studies. Finally, this study was conducted in primarily low-income Mexican-American children, which may limit its generalizability to other populations.

## Conclusion

In summary, consistent with other studies, urinary 2,4-D and two measures of OP pesticide exposure (total DMs and total DAP metabolites) were lower in children eating an organic diet. Other frequently detected metabolites for pyrethroids, diethyl OP pesticides, and the herbicide metolachlor were not significantly lower during the organic diet phase. Further, several compound-specific herbicide and OP pesticide metabolites had low detection frequencies, indicating that diet was not an important exposure source for these pesticides (e.g., diazinon, malathion) in this population. Last, independent of diet, most frequently detected metabolites were generally higher in Salinas compared with Oakland children, with DMs and metolachlor at or near significance (*p* = 0.06 and 0.03, respectively), suggesting additional sources of pesticide exposure for children living in agricultural communities. Additional research is needed to clarify the relative importance of dietary and nondietary sources of pesticide exposures in young children and determine the proportion of urinary metabolite excretion attributable to preformed metabolites.

## Supplemental Material

(730 KB) PDF

## References

[r1] Baker BP, Benbrook CM, Groth E, Lutz Benbrook K (2002). Pesticide residues in conventional, integrated pest management (IPM)-grown and organic foods: insights from three US data sets.. Food Addit Contam.

[r2] Barr DB Wilder LC Caudill SP Gonzalez AJ Needham LL Pirkle JL 2005 Urinary creatinine concentrations in the U.S. population: implications for urinary biologic monitoring measurements. Environ Health Perspect 113 192 200; 10.1289/ehp.7337 15687057 PMC1277864

[r3] Block G, Thompson FE, Hartman AM, Larkin FA, Guire KE (1992). Comparison of two dietary questionnaires validated against multiple dietary records collected during a 1-year period.. J Am Diet Assoc.

[r4] Block G, Woods M, Potosky A, Clifford C (1990). Validation of a self-administered diet history questionnaire using multiple diet records.. J Clin Epidemiol.

[r5] Bouchard MF Chevrier J Harley KG Kogut K Vedar M Calderon N 2011 Prenatal exposure to organophosphate pesticides and IQ in 7-year-old children. Environ Health Perspect 119 1189 1195; 10.1289/ehp.1003185 21507776 PMC3237357

[r6] Bradman A, Castorina R, Barr DB, Chevrier J, Harnly ME, Eisen EA (2011). Determinants of organophosphorus pesticide urinary metabolite levels in young children living in an agricultural community.. Int J Environ Res Public Health.

[r7] Bradman A Chevrier J Tager I Lipsett M Sedgwick J Macher J 2005a Association of housing disrepair indicators with cockroach and rodent infestations in a cohort of pregnant Latina women and their children. Environ Health Perspect 113 1795 1801; 10.1289/ehp.7588 16330367 PMC1314924

[r8] Bradman A Eskenazi B Barr DB Bravo R Castorina R Chevrier J 2005b Organophosphate urinary metabolite levels during pregnancy and after delivery in women living in an agricultural community. Environ Health Perspect 113 1802 1807; 10.1289/ehp.7894 16330368 PMC1314925

[r9] Bradman A Kogut K Eisen EA Jewell NP Quirós-Alcalá L Castorina R 2013 Variability of organophosphorous pesticide metabolite levels in spot and 24-hr urine samples collected from young children during 1 week. Environ Health Perspect 121 118 124; 10.1289/ehp.1104808 23052012 PMC3553429

[r10] Bradman A, Whitaker D, Quirós L, Castorina R, Claus Henn B, Nishioka M (2007). Pesticides and their metabolites in the homes and urine of farmworker children living in the Salinas Valley, CA.. J Expo Sci Environ Epidemiol.

[r11] Bravo R, Caltabiano LM, Weerasekera G, Whitehead RD, Fernandez C, Needham LL (2004). Measurement of dialkyl phosphate metabolites of organophosphorus pesticides in human urine using lyophilization with gas chromatography-tandem mass spectrometry and isotope dilution quantification.. J Expo Anal Environ Epidemiol.

[r12] CalEPA DPR (California Environmental Protection Agency, Department of Pesticide Regulation). (2006a). 2006 Annual Pesticide Use Report Indexed by Commodity: Alameda County. Sacramento, CA:CalEPA DPR.. http://www.cdpr.ca.gov/docs/pur/pur06rep/comcnty/alamed06_site.pdf.

[r13] CalEPA DPR. (2006b). 2006 Annual Pesticide Use Report Indexed by Commodity: Monterey County. Sacramento, CA:CalEPA DPR.. http://www.cdpr.ca.gov/docs/pur/pur06rep/comcnty/monter06_site.pdf.

[r14] CalEPA DPR. (2006c). Sampling for Pesticide Residues in California Well Water: 2006 Update of the Well Inventory Database For Sampling Results Reported from July 1, 2005 through June 30, 2006. EH06-05. Sacramento, CA:CalEPA DPR.. http://www.cdpr.ca.gov/docs/emon/pubs/ehapreps/eh0605.pdf.

[r15] Caudill SP, Schleicher RL, Pirkle JL (2008). Multi-rule quality control for the age-related eye disease study.. Stat Med.

[r16] Curl CL Beresford SA Fenske RA Fitzpatrick AL Lu C Nettleton JA 2015 Estimating pesticide exposure from dietary intake and organic food choices: the Multi-Ethnic Study of Atherosclerosis (MESA). Environ Health Perspect 123 475 483; 10.1289/ehp.1408197 25650532 PMC4421765

[r17] Curl CL Fenske RA Elgethun K 2003 Organophosphorus pesticide exposure of urban and suburban preschool children with organic and conventional diets. Environ Health Perspect 111 377 382; 10.1289/ehp.5754 12611667 PMC1241395

[r18] FDA (U.S. Food and Drug Administration). (2003). Total Diet Study Market Baskets 1991-3 through 2003-4.. http://www.fda.gov/downloads/Food/FoodScienceResearch/TotalDietStudy/UCM184304.pdf.

[r19] FDA. (2005). Total Diet Study: Market Baskets 2004-1 through 2005-4.. http://www.fda.gov/downloads/Food/FoodScienceResearch/TotalDietStudy/UCM291686.pdf.

[r20] Forman J, Silverstein J, Committee on Nutrition, Council on Environmental Health, American Academy of Pediatrics. (2012). Organic foods: health and environmental advantages and disadvantages.. Pediatrics.

[r21] Harnly ME, Bradman A, Nishioka M, McKone TE, Smith D, McLaughlin R (2009). Pesticides in dust from homes in an agricultural area.. Environ Sci Technol.

[r22] Hochberg Y (1988). A sharper Bonferroni procedure for multiple tests of significance.. Biometrika.

[r23] Hornung RW, Reed LD (1990). Estimation of average concentration in the presence of nondetectable values.. Appl Occup Environ Hyg.

[r24] Lu C Barr DB Pearson M Bartell S Bravo R 2006a A longitudinal approach to assessing urban and suburban children’s exposure to pyrethroid pesticides. Environ Health Perspect 114 1419 1423; 10.1289/ehp.9043 16966099 PMC1570056

[r25] Lu C Barr DB Pearson MA Waller LA 2008 Dietary intake and its contribution to longitudinal organophosphorus pesticide exposure in urban/suburban children. Environ Health Perspect 116 537 542; 10.1289/ehp.10912 18414640 PMC2290988

[r26] Lu C, Barr DB, Pearson MA, Walker LA, Bravo R (2009). The attribution of urban and suburban children’s exposure to synthetic pyrethroid insecticides: a longitudinal assessment.. J Expo Sci Environ Epidemiol.

[r27] Lu C, Bravo R, Caltabiano LM, Irish RM, Weerasekera G, Barr DB (2005). The presence of dialkylphosphates in fresh fruit juices: implication for organophosphorus pesticide exposure and risk assessments.. J Toxicol Environ Health A.

[r28] Lu C, Fenske RA, Simcox NJ, Kalman D (2000). Pesticide exposure of children in an agricultural community: evidence of household proximity to farmland and take home exposure pathways.. Environ Res.

[r29] Lu C Toepel K Irish R Fenske RA Barr DB Bravo R 2006b Organic diets significantly lower children’s dietary exposure to organophosphorus pesticides. Environ Health Perspect 114 260 263; 10.1289/ehp.8418 16451864 PMC1367841

[r30] Morgan MK, Sheldon LS, Croghan CW, Jones PA, Robertson GL, Chuang JC (2005). Exposures of preschool children to chlorpyrifos and its degradation product 3,5,6-trichloro-2-pyridinol in their everyday environments.. J Expo Anal Environ Epidemiol.

[r31] Morgan MK, Sheldon LS, Thomas KW, Egeghy PP, Croghan CW, Jones PA (2008). Adult and children’s exposure to 2,4-D from multiple sources and pathways.. J Expo Sci Environ Epidemiol.

[r32] Muñoz-Quezada MT, Lucero BA, Barr DB, Steenland K, Levy K, Ryan PB (2013). Neurodevelopmental effects in children associated with exposure to organophosphate pesticides: a systematic review.. Neurotoxicology.

[r33] Oates L, Cohen M, Braun L, Schembri A, Taskova R (2014). Reduction in urinary organophosphate pesticide metabolites in adults after a week-long organic diet.. Environ Res.

[r34] Olsson AO, Baker SE, Nguyen JV, Romanoff LC, Udunka SO, Walker RD (2004). A liquid chromatography-tandem mass spectrometry multiresidue method for quantification of specific metabolites of organophosphorus pesticides, synthetic pyrethroids, selected herbicides, and DEET in human urine.. Anal Chem.

[r35] Quirós-Alcalá L Bradman A Nishioka M Harnly ME Hubbard A McKone TE 2011 Pesticides in house dust from urban and farmworker households in California: an observational measurement study. Environ Health 10 19; 10.1186/1476-069X-10-19 21410986 PMC3071308

[r36] Quirós-Alcalá L, Bradman A, Smith K, Weerasekera G, Odetokun M, Barr DB (2012). Organophosphorous pesticide breakdown products in house dust and children’s urine.. J Expo Sci Environ Epidemiol.

[r37] Smith-Spangler C, Brandeau ML, Hunter GE, Bavinger JC, Pearson M, Eschbach PJ (2012). Are organic foods safer or healthier than conventional alternatives?: a systematic review.. Ann Intern Med.

[r38] USDA (U.S. Department of Agriculture). (2007). Pesticide Data Program. Annual Summary, Calendar Year 2006. Washington, DC:USDA.. http://www.ams.usda.gov/AMSv1.0/getfile?dDocName=STELPRDC5064786.

[r39] USDA. (2008). Pesticide Data Program. Washington, DC:USDA.. http://www.ams.usda.gov/datasets/pdp.

[r40] USDA. (2013). Pesticide Data Program. Annual Summary, Calendar Year 2011. Washington, DC:USDA.. http://www.ams.usda.gov/AMSv1.0/getfile?dDocName=stelprdc5102692.

[r41] U.S. EPA (U.S. Environmental Protection Agency). (2000). Chlorpyrifos. Revised risk assessment and agreement with registrants.. Fed Reg.

[r42] U.S. EPA. (2001). Diazinon revised risk assessment and agreement with registrants.. Fed Reg.

[r43] Whyatt RM, Camann DE, Kinney PL, Reyes A, Ramirez J, Dietrich J (2002). Residential pesticide use during pregnancy among a cohort of urban minority women.. Environ Health Perspect.

[r44] Wilson NK, Chuang JC, Lyu C, Menton R, Morgan MK (2003). Aggregate exposures of nine preschool children to persistent organic pollutants at day care and at home.. J Expo Anal Environ Epidemiol.

[r45] Zhang X, Driver JH, Li Y, Ross JH, Krieger RI (2008). Dialkylphosphates (DAPs) in fruits and vegetables may confound biomonitoring in organophosphorus insecticide exposure and risk assessment.. J Agric Food Chem.

