# Supplementary material for: Effect of Organic Diet Intervention on Pesticide Exposures in Young Children Living in Low-Income Urban and Agricultural Communities
Source: Environ Health Perspect. 2015 Apr 10;123(10):1086–93. doi: 10.1289/ehp.1408660 (PMC4590750; doi:10.1289/ehp.1408660)
Supplement: (730 KB) PDF [file ehp.1408660.s001.acco.pdf]

Environ Health Perspect
doi: 10.1289/ehp.1408660

Note to Readers: *EHP* strives to ensure that all journal content is accessible to all readers. However, some figures and Supplemental Material published in *EHP* articles may not conform to 508 standards due to the complexity of the information being presented. If you need assistance accessing journal content, please contact ehp508@niehs.nih.gov. Our staff will work with you to assess and meet your accessibility needs within 3 working days.

Supplemental Material

Effect of Organic Diet Intervention on Pesticide Exposures in Young Children Living in Low-Income Urban and Agricultural Communities

Asa Bradman, Lesliam Quirós-Alcalá, Rosemary Castorina, Raul Aguilar Schall, Jose Camacho, Nina T. Holland, Dana Boyd Barr, and Brenda Eskenazi

Table of Contents

Table S1. Frequency of food consumption in all children by diet phase (N=40 children).[a]

Table S2. Personal characteristics of study participants (N=40).

Table S3. Summary statistics for frequently detected urinary metabolites for participating children by conventional and organic diet phase.

Table S4. Detection frequency by diet phase (conventional vs. organic) for metabolites with low overall detection frequencies.

Table S5. Estimated effect of an organic diet (vs. conventional) on the geometric mean for frequently detected metabolites using creatinine-adjusted urinary concentrations.[a]

Figure S1. Estimated marginal adjusted GMs and confidence intervals for select urinary metabolites based on diet followed after fitting of linear mixed-effects models (creatinine-

adjusted). All models were adjusted for type of void (FMV vs. random spot sample). Models for "All children" were also adjusted for location (Oakland vs. Salinas); an interaction term for location and diet was included in these models for total DEs and 3-PBA ($p_{int} \leq 0.20$). P-values reported in the figures indicate whether there were significant differences observed in creatinine-adjusted metabolite concentrations between diet phases by location. P-values reported at the bottom of the figures indicate significance for the difference of creatinine-adjusted metabolite concentrations between locations irrespective of diet.

Table S1. Frequency of food consumption in all children by diet phase (N=40 children).[a]

**Food group and diet phase**	**Average # times definitely eaten/day(SD)**	**Average # times definitely and probably eaten/day(SD)**	**P-value[b] (definitely eaten / definitely and probably eaten)**
Beans			
C1	1.1 (0.17)	1.1 (0.16)	
Org	1.1 (0.27)	1.1 (0.27)	0.422 / 0.430
C2	1.1 (0.17)	1.1 (0.20)	
Grains			
C1	3.9 (1.15)$^{\alpha}$	4.0 (1.17)$^{\beta}$	
Org	3.3 (1.18)$^{\alpha}$	3.5 (1.15)$^{\beta}$	0.032 / 0.024
C2	3.5 (1.12)	3.7 (1.06)	
Fruits			
C1	2.6 (1.09)$^{\gamma}$	3.1 (1.28)	
Org	3.2 (1.57)$^{\delta}$	3.7 (1.57)$^{\varepsilon}$	0.001 / 0.006
C2	2.3 (1.55)$^{\gamma\delta}$	2.7 (1.55)$^{\varepsilon}$	
Vegetables			
C1	1.7 (0.56)	2.2 (0.73)	
Org	1.8 (0.60)	2.3 (0.73)	0.482 / 0.599
C2	1.6 (0.55)	2.2 (0.69)	
Meats			
C1	1.6 (0.39)	1.8 (0.45)$^{\lambda}$	
Org	1.4 (0.34)	1.5 (0.40)$^{\lambda\mu}$	0.059 / 0.003
C2	1.5 (0.44)	1.7 (0.44)$^{\mu}$	
Dairy			
C1	3.4 (0.97)	4.4 (1.28)	
Org	3.5 (1.53)	4.3 (1.84)	0.568 / 0.502
C2	3.3 (1.34)	4.3 (1.62)	

[a]Daily food frequency diaries were used to arrive at the values reported. Each food item/dish ingredient reported was grouped into different food groups (e.g., beans, grains, fruits, vegetables, meats, and dairy) to estimate the average number of times they definitely or probably consumed that item based on what was reported. An item was assigned a category of "probably consumed" if it was suspected of being an ingredient based on the dish reported. [b]Kruskal-Wallis test adjusted for ties. Null hypothesis: no difference in median frequency of consumption for all children across both conventional diet phases and the organic diet phase. Alternative hypothesis: at least one group is different from the others.

Note: When Kruskal-Wallis was rejected, pairwise Wilcoxon rank sum test were performed.

The paired Greek letters indicate which 2 phases are significantly different (Wilcoxon rank sum $p<0.05$) as follows:

α indicates that "Average # times definitely eaten/day" of grains is significantly different in C1 compared to Org; β indicates that "Average # times definitely and probably eaten/day" of grains is significantly different in C1 compared to Org; γ indicates that "Average # times definitely eaten/day" of fruits is significantly different in C1 compared to C2; δ indicates that "Average # times definitely eaten/day" of fruits is significantly different in Org compared to C2; ε indicates that "Average # times definitely and

probably eaten/day" of fruits is significantly different in Org compared to C2; λ indicates that "Average # times definitely and probably eaten/day" of meats is significantly different in C1 compared to Org; and μ indicates that "Average # times definitely and probably eaten/day" of meats is significantly different in Org compared to C2.

Table S2. Personal characteristics of study participants (N=40).

Characteristic	Oakland (n=20)	Salinas (n=20)
	n (%)	n (%)
Gender		
Boys	10 (50%)	9 (45%)
Girls	10 (50%)	11 (55%)
Poverty Level		
≤ poverty threshold	13 (65%)	13 (65%)
>poverty threshold	7 (35%)	7 (35%)
Marital Status		
Married or living as married	19 (95%)	19 (95%)
Separated	--	1 (5%)
Widowed	1 (5%)	--
Mother currently works in farmwork		
No	20 (100%)	16 (80%)
Yes	--	4 (20%)
Father works in ag or related profession with potential exposure to pesticides[a]		
No	20 (100%)	1 (5%)
Yes	--	18 (95%)
Distance to nearest ag field or golf course		
50-200 ft.	--	1 (5%)
200 ft. to ≤ ¼ mile	--	3 (15%)
> ¼ mile	20 (100%)	16 (80 %)
Child has at least one farmworker parent		
No	20 (100%)	18 (90%)
Yes	--	2 (10%)[a]
Reported pesticide use during the study period		
No	14 (70%)	17 (85%)
Yes	6 (30%)	3 (15%)
Current pesticide use (within last 3 months) reported at baseline		
No	7 (35%)	14 (70%)
Yes[b]	13 (65%)	6 (30%)
Pesticides used during the study in the home		
Cypermethrin/Imiprothrin formulation	3 (15%)	1 (5%)
Allethrin/Sumithrin[c] formulation	1 (5%)	--
Eugenol/phenythyl propionate	1 (5%)	--
Boric Acid	--	1 (5%)
Unknown/Professional Application	2 (10%)	1 (5%)

[a]For Salinas children: one participant had a father who worked in agriculture in the 3 months preceding the study, but not during study period. This participant's information/measurements were still included in our analyses. Another participant indicated that they lived with a farmworker, but there was no evidence of this during the study period. Inclusion/exclusion of these children did not change our results. [b]Most of the insecticides used were pyrethroid formulations. [c]Sumithrin is also known as d-Phenothrin.

Table S3. Summary statistics for frequently detected urinary metabolites for participating children by conventional and organic diet phase.

Metabolite, Population, Diet Phase	n	DF (%)	GM (GSD)	p50	Max
**Total DMs (nmol/L)**					
All (n=40)					
C1	159	96	105.4 (3.8)*	95.2	7718.0
Org	168	88	54.1 (4.3)	53.4	2618.6
C2	71	93	112.9 (5.0)*	92.4	6346.5
Oakland (n=20)					
C1	81	94	81.2 (3.6)	76.4	2435.0
Org	83	93	50.3 (3.7)	51.6	1988.7
C2	85	94	80.9 (4.4)	59.7	3564.8
Salinas (n=20)					
C1	78	99	138.2 (3.8)*	131.0	7718.0
Org	85	84	58.2 (4.9)	58.2	2618.6
C2	37	92	153.5 (5.3)[†]	182.7	6346.5
**Total DEs (nmol/L)**					
All (n=40)					
C1	159	75	19.4 (5.3)	24.8	1408.9
Org	168	71	18.4 (5.9)	23.1	470.3
C2	71	77	20.1 (5.1)	26.3	529.8
Oakland (n=20)					
C1	81	80	18.9 (4.4)	23.0	412.4
Org	83	82	25.3 (4.9)	28.0	448.8
C2	84	85	18.5 (3.2)	21.6	108.4
Salinas (n=20)					
C1	78	71	20.0 (6.4)	27.9	1408.9
Org	85	61	13.4 (6.7)	18.0	470.3
C2	37	70	21.6 (7.1)	30.2	529.8
**Total DAPs (nmol/L)**					
All (n=40)					
C1	159	97	149.6 (3.4)[†]	143.8	8031.1
Org	168	92	90.2 (4.0)	97.2	2711.0
C2	71	97	164.7 (4.0)[†]	137.7	6463.5
Oakland (n=20)					
C1	81	96	118.8 (3.2)	112.1	2480.4
Org	83	98	91.4 (3.3)	93.7	2138.7
C2	34	97	122.6 (3.4)	91.1	3585.0
Salinas (n=20)					
C1	78	99	190.1 (3.6)[†]	171.0	8031.1
Org	85	87	88.9 (4.8)	97.5	2711.0
C2	37	97	216.0 (4.5)	218.0	6463.5
**MET (ng/mL)**					
All (n=40)					
C1	155	70	0.2 (1.8)	0.2	0.6
Org	152	69	0.2 (1.8)	0.2	0.6
C2	71	82	0.2 (1.6)	0.2	0.3
Oakland (n=20)					
C1	81	67	0.2 (1.8)	0.2	0.3
Org	83	64	0.1 (1.8)	0.2	0.3

Metabolite, Population, Diet Phase	n	DF (%)	GM (GSD)	p50	Max
C2	34	71	0.2 (1.7)	0.2	0.3
Salinas (n=20)					
C1	74	73	0.2 (1.9)	0.2	0.6
Org	69	75	0.2 (1.8)	0.2	0.6
C2	37	92	0.2 (1.4)	0.2	0.3
**2,4-D (ng/mL)**					
All (n=40)					
C1	155	88	0.4 (2.6)	0.4	6.8
Org	152	88	0.3 (2.1)	0.3	1.9
C2	71	100	0.4 (2.0)	0.4	3.0
Oakland (n=20)					
C1	81	93	0.3 (2.1)	0.3	1.4
Org	83	93	0.3 (1.9)	0.3	1.1
C2	34	100	0.4 (1.7)	0.3	1.6
Salinas (n=20)					
C1	74	84	0.4 (3.1)	0.4	6.8
Org	69	83	0.3 (2.4)	0.3	1.9
C2	37	100	0.5 (2.1)	0.5	3.0
**3-PBA (ng/mL)**					
All (n=40)					
C1	137	79	0.6 (3.1)	0.6	27.7
Org	126	90	0.6 (2.1)	0.6	8.2
C2	68	72	0.5 (3.1)	0.6	30.3
Oakland (n=20)					
C1	78	99	0.9 (2.3)	0.8	24.0
Org	78	92	0.6 (2.1)	0.7	3.7
C2	33	82	0.8 (2.9)	0.8	8.7
Salinas (n=20)					
C1	59	53	0.4 (3.6)	0.3	27.7
Org	48	85	0.5 (2.1)	0.6	8.2
C2	35	63	0.4 (2.9)	0.3	30.3

Abbreviations: C1=Conventional diet phase 1; Org=Organic diet phase; C2=Conventional diet phase 2; DF=Detection frequency; GM (GSD): Geometric Mean (Geometric Standard Deviation).

*,† Indicates whether mean metabolite concentrations in the respective conventional diet phase was significantly different from the adjacent organic diet phase. Significance was adjusted for multiple testing using the Hochberg procedure where *= $p \leq 0.001$ and † = $0.001 < p \leq 0.005$.

Table S4. Detection frequency by diet phase (conventional vs. organic) for metabolites with low overall detection frequencies.

**Exposure**	**Conv**	**Org**
**ORGANOPHOSPHORUS INSECTICIDES**		
Precursor: Coumaphos<br>Metabolite: 3-chloro-4-methyl-7-hydroxycoumarin (CMH)		
n	226	152
DF (%)	23	24
Precursor: Diazinon<br>Metabolite: 2-isopropyl-4-methyl-6-hydroxypyrimidin (IMPY)		
n	230	168
DF (%)	20	27
Precursor: Pirimiphos-methyl<br>Metabolite: 2-diethylamino-6-methyl pyrimidin-4-ol (DPY)		
n	230	168
DF (%)	4	2
Precursor: Isazophos<br>Metabolite: 5-chloro-1,2-dihydro-1-isopropyl-[3H]-1 (CIT)		
n	230	168
DF (%)	31	30
Precursor: Malathion<br>Metabolite: malathion dicarboxylic acid (MDA)		
n	226	152
DF (%)	42*	32
**PYRETHROID INSECTICIDES**		
Precursor: Allethrin, phenothrin, pyrethrum, resmethrin, tetramethrin<br>Metabolite: chrysanthemum dicarboxylic acid (CDCA)		
n	217	134
DF (%)	ND	ND
Precursor: Cyfluthrin<br>Metabolite: 4-fluoro-3-phenoxybenzoic acid (4FP)		
n	217	136
DF (%)	33*	21
Precursor: Deltamethrin<br>Metabolite: cis-2,2-(dibromo)-2-dimethylvinylcyclopropane carboxylic acid (DBCA)		
n	226	152
DF (%)	1.3	ND
Precursor: cis-cypermethrin, cis-cyfluthrin, cis-permethrin<br>Metabolite: cis-2,2-(dichloro)-2-dimethylvinylcyclopropane carboxylic acid (*cis*-DCCA)		

Exposure	Conv	Org
n	220	149
DF (%)	0.9	0.7
Precursor: trans-cypermethrin, trans-cyfluthrin, trans-permethrin<br>Metabolite: trans-2,2-(dichloro)-2-dimethylvinylcyclopropane carboxylic acid (*trans*-DCCA)		
n	220	149
DF (%)	7	7
**HERBICIDES**		
Precursor: 2,4,5-trichlorophenoxyacetic acid<br>Metabolite: 2,4,5-trichlorophenoxyacetic acid (2,4,5T)		
n	226	152
DF (%)	24	21
Precursor: Acetochlor<br>Metabolite: acetochlor mercapturate (ACE)		
n	230	168
DF (%)	7	7
Precursor: Alachlor<br>Metabolite: alachlor mercapturate (ALA)		
n	230	168
DF (%)	26	24
Precursor: Atrazine<br>Metabolite: atrazine mercapturate (ATZ)		
n	230	168
DF (%)	ND	ND

Abbreviations: Conv: Samples collected during the conventional diet phases; Org.: Samples collected during the organic diet phase; ND: Not detected.

*=$p<0.05$, where p-value indicates whether there is a statistically significant difference in detection between diet phases (Chi-square test).

Table S5. Estimated effect of an organic diet (vs. conventional) on the geometric mean for frequently detected metabolites using creatinine-adjusted urinary concentrations.[a]

Children	Total DEs	Total DMs	Total DAPs	MET	2,4-D	3-PBA
**All, n=40**						
% Change (95% CI)	12.1 (-25.8, 69.6)	-41.6 (-60.1, -14.6)	-31.6 (-51.9, -2.6)	6.3 (-10.1, 25.7)	-15.2 (-30.5, 3.4)	-4.9 (-21.7, 15.3)
p-value	0. 587	0.005	0.035	0.475	0.102	0.607
**Oakland, n=20**						
% Change (95% CI)	48.3 (-21.2, 179.1)	--	--	--	--	-28.8 (-44.1, -9.2)
p-value	0.222					0.006
**Salinas, n=20**						
% Change (95% CI)	-15.0 (-50.0, 44.7)	--	--	--	--	39.5 (2.4, 90.1)
p-value	0.550					0.035
$p_{\text{-interaction}}$	0.186	0.512	0.313	0.259	0.480	0.001

Abbreviations: CI= confidence interval.

[a]Marginal results by location are omitted if observed interaction between location and diet was not significant (p>0.20). In these cases only, the model without the interaction term is presented for all children.

Figure S1.

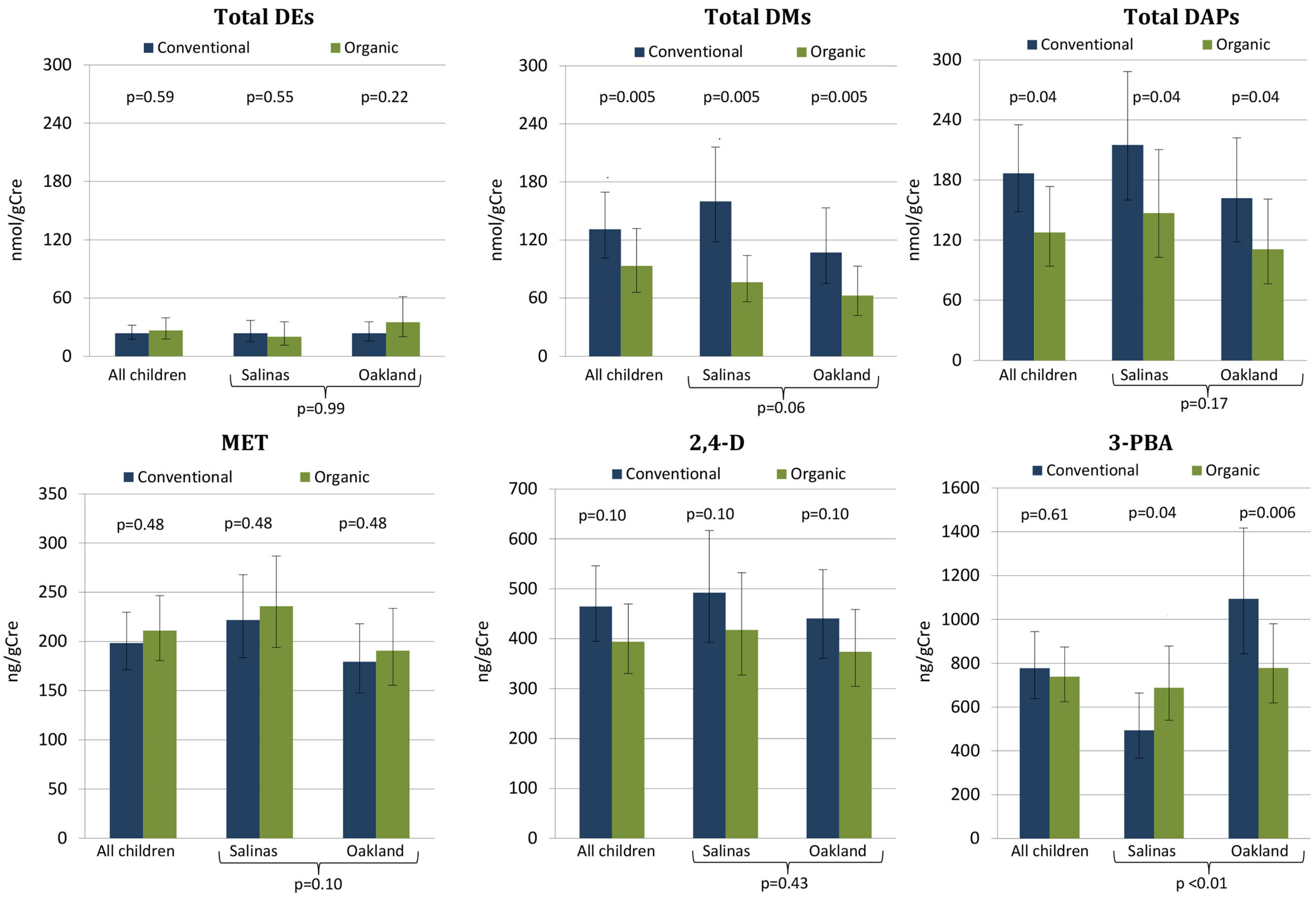
Total DEs
Conventional
Organic
nmol/gCre
300
240
180
120
60
0
p=0.59
p=0.55
p=0.22
All children
Salinas
Oakland
p=0.99
Total DMs
Conventional
Organic
nmol/gCre
300
240
180
120
60
0
p=0.005
p=0.005
p=0.005
All children
Salinas
Oakland
p=0.06
Total DAPs
Conventional
Organic
nmol/gCre
300
240
180
120
60
0
p=0.04
p=0.04
p=0.04
All children
Salinas
Oakland
p=0.17
MET
Conventional
Organic
ng/gCre
350
300
250
200
150
100
50
0
p=0.48
p=0.48
p=0.48
All children
Salinas
Oakland
p=0.10
2,4-D
Conventional
Organic
ng/gCre
700
600
500
400
300
200
100
0
p=0.10
p=0.10
p=0.10
All children
Salinas
Oakland
p=0.43
3-PBA
Conventional
Organic
ng/gCre
1600
1400
1200
1000
800
600
400
200
0
p=0.61
p=0.04
p=0.006
All children
Salinas
Oakland
p <0.01

Figure S1. Estimated marginal adjusted GMs and confidence intervals for select urinary metabolites based on diet followed after fitting of linear mixed-effects models (creatinine-adjusted). All models were adjusted for type of void (FMV vs. random spot sample). Models for "All children" were also adjusted for location (Oakland vs. Salinas); an interaction term for location and diet was included in these models for total DEs and 3-PBA ($p_{int} \leq 0.20$). P-values reported in the figures indicate whether there were significant differences observed in creatinine-adjusted metabolite concentrations between diet phases by location. P-values reported at the bottom of the figures indicate significance for the difference of creatinine-adjusted metabolite concentrations between locations irrespective of diet.
